# Effects of AR Picture Books on German Teaching in Universities

**DOI:** 10.3390/jintelligence10010013

**Published:** 2022-02-14

**Authors:** Chao Gu, Jiangjie Chen, Chun Yang, Wei Wei, Qianling Jiang, Liao Jiang, Qiuhong Wu, Shu-Yuan Lin, Yunshuo Yang

**Affiliations:** 1 Department of Culture and Arts Management, Honam University, Gwangju 62399, Korea; cguamoy@my.honam.ac.kr; 2 School of Design, Jiangnan University, Wuxi 214122, China; d10717016@ms.ttu.edu.tw (J.C.); 8202201014@jiangnan.edu.cn (C.Y.); jiangqianling@jiangnan.edu.cn (Q.J.); 3 School of Textile, Garment, and Design, Changshu Institute of Technology, Changshu 215500, China; doublewei@cslg.edu.cn; 4 School of art and design, Minnan Science and Technology University, Quanzhou 362300, China; jiangliao@mku.edu.cn (L.J.); qhwu363@gmail.com (Q.W.); 5 Department of Media Design, Tatung University, Taipei 104, Taiwan; shuyuan@gm.ttu.edu.tw; 6 College of Foreign Languages and Cultures, Xiamen Universtiy, Xiamen 361005, China

**Keywords:** augmented reality, cooperative learning, media in education, teaching strategies

## Abstract

In this paper, we discuss the teaching effects of augmented reality (AR) technology in German instruction. We conducted one prestudy and three formal studies on German learners in China’s mainland and Taiwan region. In the formal studies, a total of 120 students participated in the survey, allowing us to compare the differences in interest in learning between AR picture books and traditional picture books. A total of 114 students took part in the survey, which enabled us to compare the contribution of AR picture books to teaching when students’ satisfaction and German proficiency were different. To improve satisfaction, 514 students participated in the survey regarding the influence of the interactive narrative design effect and peer learning on satisfaction with using AR picture books. The results suggest that when learning German with AR picture books, satisfaction is the key construct that determines students’ learning states.

## 1. Introduction

The process of learning German easily falls into two extreme cycles of rejection after setbacks and the accumulation of achievement and motivation (Noels et al. 2016). Being in the former position can make the learner feel resistant to studying, while being in the latter position can provide additional help as learning progresses. How to avoid going to negative extremes underscores the importance of psychological research in German instruction. Teachers need to think more about how students’ internal motivation and external behaviour evolve over time, as well as other psychological and environmental factors that may influence changes in students’ mentality (Dörnyei et al. 2014). In German instruction, we should try to help students build positive emotions to achieve good learning outcomes. This requires the constant review and iteration of teaching methods. In German teaching, teachers need to try new teaching methods as well as evaluate existing ones. Choosing more effective teaching methods can help improve the quality and popularity of education.

At present, there are some commonly discussed teaching methods in foreign language teaching. Canadian educators have proposed the immersion teaching method (ITM) for writing and foreign language teaching (Zhong and Zhu 2019). The ITM involves placing students in a linguistic environment other than that of their mother tongue and teaching them entirely in a foreign language (Li 2021). By encouraging students to adapt to the foreign language they wish to learn and to receive more language training, students can practice their own language expression level (Ye 2021). Communicative language teaching (CLT) is aimed at improving communicative competence (Richards and Schmidt 2013). CLT’s focus is no longer limited to established listening and speaking training but encourages students to use language as a meaningful communication tool. CLT creates opportunities for learners to communicate in a foreign language and to integrate communication activities into a broader language teaching programme (Howatt 1984). Task-based language teaching (TLT) emphasises ‘learning by doing’. TLT was developed from CLT and centres on student expression (Ellis 2016). Nunan (1989) proposed the framework of TLT, including teaching objectives, information input, activities, the roles of teachers and students, teaching situations, and other content. Before using TLT, teachers should identify students’ needs and design tasks according to those needs so that students can better apply linguistic knowledge to real life (Long 2014). In TLT instruction, it is necessary to create a situation in which students continuously express their ideas in the target language and achieve a degree of proficiency that others can comprehend (Prabhu 1987). The grammar-translation method (GTM) is a classic teaching approach where one learns a language through the detailed analysis of grammatical rules and carries out constant translation between the target language and one’s mother tongue (Richards and Rodgers 2014). VanPatten (2003) asserted that one of the main goals of GTM is to cultivate students’ reading and translation abilities through memorising grammar rules and completing grammar exercises; another goal is to cultivate students’ comprehensive intelligence. Despite the variety of language teaching methods available, they are still essentially based in offline classrooms. With the rapid development of digital media technology, digital education has brought new possibilities for language learning. Exploring new teaching methods based on digital media technology may help establish a more scientific and ideal foreign language learning process and help students acquire foreign languages more effectively.

We focus on the teaching effect of AR picture books on students’ reading in German instruction. Picture books are a common means of foreign language learning. As a carrier of high contextual diversity, they can play a better role in learning by narrating stories with pictures (Damayanti and Febrianti 2020). The use of picture books is obviously helpful to foreign language learning and is critical to the entire process. Although many scholars oppose the use of translation as a learning strategy in classroom teaching, continuous translation between the targeted foreign language and one’s mother tongue during the reading process is similar to a natural response of language learners (Cook 2007). The pauses in translation caused by unfamiliar words and sentences in reading paragraphs often make it difficult for learners to concentrate on and grasp knowledge points, resulting in low efficiency in learning German by reading textbooks. This has a negative impact on students’ mindset. Therefore, marking in teaching text paragraphs is vital and helps students clarify the focus of learning and reduce the impact of new words (Degand and Sanders 2002). Content that is more likely to be recalled after reading is the bold or specially marked part of the paragraph, rather than content that is not clearly indicated in typesetting (Peters 2012). Text marking is used to distinguish relatively important content in a paragraph to help readers notice more relevant text (Hyuk 2003). Geva (1986) pointed out that students performed better when reading bold, marked text than when reading unmarked text. Geva and Ryan (1985) found that students’ reading comprehension improved after highlighting the typesetting of key content in the text. Appropriate labelling helps language learners read the text and reduces negative perceptions by reducing the frequency of incomprehension. Therefore, it is urgent to test innovative marking approaches for picture books. Much of the existing research has focused on how to mark texts more scientifically (Bishop 2004; De Ridder 2002), but marking instructional texts in German picture books through AR interaction has not yet been discussed.

The most salient feature of AR technology is tracking. Virtual objects’ movements can be tracked by calculating detected features. Artificial markers are widely used (Brito and Stoyanova 2018) to display virtual objects in German instruction. This paper focuses on three research questions to verify the significance of using AR picture books in German teaching and how to design AR picture books that are more conducive to German teaching. Firstly, compare the respective teaching effects of AR picture books and traditional picture books. If traditional picture books are introduced into AR picture books, better teaching effects will be achieved, indicating that extra production costs are worth paying. Secondly, through the comparison of learning interests (LI), continuous learning intention (CLI), and learning outcomes (LO); it is tested whether students’ satisfaction with AR picture books will have an impact on learning. This is closely tied to the design of picture books. If there is a difference in teaching effects between picture books with different satisfaction levels, it represents the significance of studying students’ preferences for AR picture books. Thirdly, the structural equation model is established to put forward suggestions for the design of AR picture books. AR picture books present text in an interactive way, so the design effects of interactive narratives and flow experience need to be discussed. In addition, the effect of peer learning needs to be examined because of its possible influence on students’ learning processes. Unlike traditional picture books, AR picture books can present content in an interactive way. Given that AR technology can help teaching in nature, music, and many other subjects (Huang et al. 2011; Kerawalla et al. 2006; Wei et al. 2015), it may also be helpful in German instruction at universities. At present, the important growth markets for German learning are in Asia, especially China. According to data surveys conducted by the German Ministry of Foreign Affairs and other institutions, about 145,000 people studied German in China in 2020, reaching a 25% increase compared to 109,000 in 2015 (Goetheinstitut 2020). In 2018, China’s Ministry of Education announced that German and other minor languages would be included in the national college entrance examination, further confirming the importance of German learning. Therefore, this paper chooses students from China’s mainland and Taiwan region as research objects to investigate the influence of AR picture books on their German learning. It is an innovative mode of thinking to educate with interactivity. It is critical to study the application of AR picture books in German instruction. The application of AR picture books in German education has promoted the theoretical development of new pedagogical techniques. In terms of management significance, the contribution of AR picture books to instruction can provide a sufficient reason for schools and teachers to use AR picture books to teach German, and students’ user behaviour in the learning process provides an aesthetic basis for designers. Although some educational scholars have paid attention to the help of AR technology in language education, there is still no comprehensive quantitative evaluation of AR picture books in specific language teaching. Considering that it is important and urgent to improve the teaching effect in German learning, this paper has theoretical and practical novelty and contribution.

## 2. Literature Review

### 2.1. The Application of AR Technology

AR technology superimposes digital images on the real environment to present a different world (Furht 2011). Using AR technology, virtual objects seem to be part of the real world. AR technology has the following two important simultaneous features: interactivity in real time and registering an object in three dimensions (Azuma 1997). Virtual objects are added to the real environment in real time during the user experience (Cipresso et al. 2018). Users are at the intersection of virtual and real users in the AR environment. The technology is based on the following three requirements: tracking real-world objects, processing information, and presenting the synthesised information to the user (Carmigniani et al. 2011). Handheld devices are one of the most common ways to achieve AR. Users can hold up the device to obtain a sensory experience of the world after a digital overlay (Giunta et al. 2018).

The application of AR in education is of great significance. AR not only makes mobile learning more possible, but also offers students different learning experiences in e-learning environments (Arici et al. 2019). Students perceive meaningful interactions when AR is used to teach French (Perry 2015). Through visual and auditory experiences different from traditional teaching, instruction in AR scenarios is beneficial for teachers to convey knowledge points and to effectively receive student feedback (Soltani and Morice 2020). AR has become a hot topic in education in recent years (Ibáñez and Delgado-Kloos 2018). Radu (2014) reviewed 26 publications and pointed out that AR technology can promote cooperation among students and improve their motivation to learn. Akçayır and Akçayır (2017) reviewed 68 research articles on the application of AR technology in teaching and affirmed that AR technology is beneficial for solving educational problems such as insufficient classroom time, classroom congestion, and teachers’ inexperience. AR technology is conducive to boosting students’ motivation, satisfaction, attention, engagement, and enjoyment (Parmaxi and Demetriou 2020). Possible reasons include the newness of the experience, the welcoming opportunity, or the teachers allowing students to move around the classroom (Godwin-Jones 2016). The promotion of AR in education may come from its media characteristics, such as sensory immersion, navigation, and manipulation (Cheng and Tsai 2013). These features foster students’ positive emotions and help them achieve their learning goals efficiently (Wu et al. 2013).

### 2.2. Multimedia Learning

#### 2.2.1. Interactive Narrative Design Effect (INDE)

Interactive narratives are a subject of concern in multimedia research (Si et al. 2005). Guiding students to reach their instructional goals through interactive narrative training can improve their educational experience and boost their learning (Riedl and Bulitko 2012). Interactive narratives have been employed in science, technology, engineering, math (Rowe et al. 2011), and military education (Zook et al. 2012). Interactive narratives can contribute to education by focusing on the user (Mott et al. 2006). Because AR technology can present text in the form of digital media, applications with interactive narratives are beginning to explore the possibility of combining with AR (Sezen et al. 2020).

**Figure 1:**
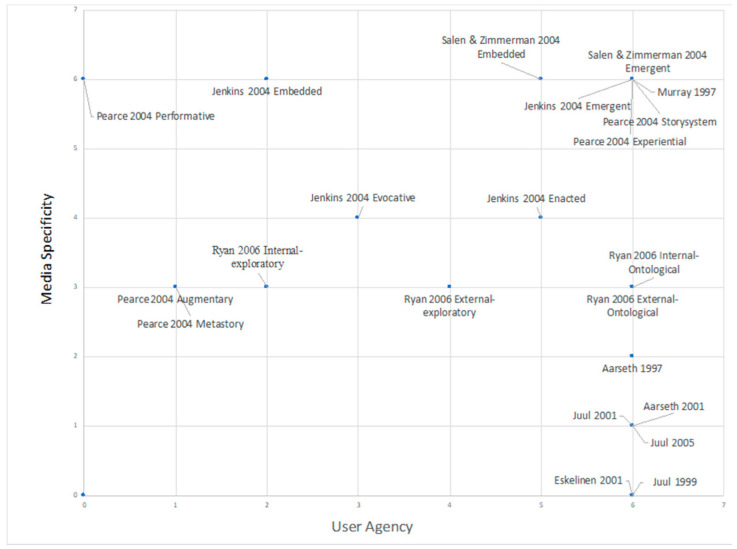
Spatial mapping of different ontological positions onto an interactive narrative (Koenitz 2018).

Koenitz (2018) asserted that AR is a multimedia platform that can interact with users and present content in an interactive narrative. To explore the influence of interactive narratives, the relationship between the user’s influence on multimedia (user agency) and the degree of influence of narratives through multimedia (media specificity) was mapped onto a 2D image after the integration of 11 interactive narrative-related studies (E. Aarseth 2001; E. J. Aarseth 1997; Eskelinen 2001; Henry 2004; Juul 1999, 2001, 2011; Murray 2017; Pearce 2004; Ryan 2006; Salen et al. 2004). As shown in Figure 1, the values of 0–7 correspond to the degree of enhancement. The outcome is a space for debate, and researchers with different positions have different views on the relationship between the two. This suggests that interactive narratives might alter the results of text presentation, but the extent of such a change remains uncertain. Previous studies have attempted to develop interactive narratives of AR picture books to encourage young people to protect animals (Dionisio et al. 2020). The combination of AR and interactive narratives has also been used to promote tourism and cultural sites to improve the user experience (La Scala et al. 2016). What is exciting is that participants who engaged in AR games for moral education and absorbed the educational content through interactive narratives generated positive feedback after participating in the games (Jin et al. 2020). This indicates that interactive narratives, designed to mesh with AR, may be helpful to education. It is vital to examine the influence of the interactive narrative features of AR picture books on German instruction.

#### 2.2.2. The Flow Model

Flow theory is one of the most important theories in multimedia learning. Finneran and Zhang (2003) pointed out in the PAT model of flow that person, task, and artefact, as antecedents, jointly lead to changes in users’ flow experiences. In addition, they may cause shifts in terms of consequences. This suggests that students’ willingness may be affected by changes in flow antecedents. The importance of flow antecedents has been verified in many studies. As for tasks, the comparison of students’ learning effects in different task groups indicates that adjusting the difficulty of tasks can impact students’ flow experience (Kawabata 2018). Regarding the person, students’ self-regulation of their emotional experiences in the online learning process affects flow experience, and students’ self-discipline has a positive impact on flow (Wan et al. 2020). For artefacts, previous studies have compared participants learning with AR to reveal higher levels of flow experience than participants using web-based applications (Ibáñez et al. 2014). The use of AR in German instruction might not only be influenced by the person factors associated with students, but also affect students’ flow experience due to different artefacts from traditional textbooks and changes in tasks that students need to solve during the learning process. In view of the fact that flow experience has a promoting effect on learning interest (Guo et al. 2016) and a helping effect on improving continuous intention (Guo et al. 2012), the application of AR technology in German instruction may ultimately bring about different intentions due to modifying students’ flow experience.

#### 2.2.3. Peer Learning in Multimedia

Peer learning refers to interacting with one’s counterparts from the same social group who are not professional teachers and who help each other gain knowledge and skills (Topping 2005). The concept can be further subdivided into the following two situations: The first involves a one-to-one relationship of encouragement and support established by a more experienced person, known as peer tutoring. The second scenario entails cooperative learning where students pursue common goals and outputs by building positive interdependence (Slavin 1991). Peer learning relationships are widely used in education (Fuchs et al. 1997; Mathes et al. 1998) and to enhance university instruction (Topping 1998). A study of college students’ programming skills showed that students who studied in pairs wrote higher-quality code than those who programmed alone (Demir and Seferoglu 2021). Peer learning is highly practical and may improve students’ learning outcomes (Boud et al. 1999). Some studies have proven the effectiveness of peer learning when students harness multimedia for learning. For example, the use of multimedia-assisted English instruction with peer support can boost students’ oral expression in foreign language learning (Muslem and Abbas 2017). When students employ multimedia to acquire native language vocabulary, peer relationships are beneficial to mastery (Silverman et al. 2017). In view of the importance of peer learning in multimedia instruction, it is meaningful to test the effect of peer relationships when applying AR technology to learning German.

### 2.3. Using AR Picture Books to Teach German

Learning German is inseparable from interactions and communication in the social context (Bongartz and Schneider 2003). Simple, decontextualised learning makes it difficult for students to acquire knowledge and abilities mastered in a specific context. Text reading is important in language learning because it imitates situations of multiple social interactions. However, improvements in reading and spelling might not occur at the same pace. As shown by Scheerer-Neumann et al. (1986), some learners can spell correctly but cannot accurately read and understand what they have already spelled. This indicates that although reading and spelling are the two linguistic abilities that need to be considered simultaneously in German learning, there may be differences in the level of the two in terms of practical application (Valtin 1989).

In Germany, teachers pay close attention to phonetics in German teaching (Valtin 1997). Unlike many English words where the same vowel sounds are pronounced differently, German pronunciation contains a simple, direct grapheme-phoneme correspondence (Wimmer 1996). Although this seems easier to master, German still manifests a pattern of word-final obstruent devoicing and many features that need to be considered (Charles-Luce 1985). Hence, learning a second foreign language may be influenced by the language rules of one’s mother tongue (Smith and Peterson 2012). Long-term pronunciation and listening training can help learners achieve an accent that is closer to standardisation. For example, approximately 60% of native German speakers can perceive the proportion of minor sound pause differences between the preceding vowel duration and consonant closure duration (Port and O’Dell 1985).

It is crucial to guide students’ attention to reading texts through language teaching exploration and teaching method design and to combine reading, spelling, listening, and speaking organically. This requires teachers to help students choose suitable learning methods in a variety of categories. Shimizu and Ogawa (2014) showed that in short-term language learning, reading on an electronic screen may have a more positive effect on word memory than reading on a handwritten map card. However, the positive and negative effects are strongly influenced by the learner’s own attributes and learning plans. This proves that the choice of learning technique or the degree of using the method may directly affect the learning outcome (Lin 2009). It also corresponds to the pattern of human, equipment, and task factors working together in flow theory (Finneran and Zhang 2003). Unlike directly relying on electronic media to display text, AR picture books add interactive virtual objects in the context of a real environment. AR can enhance students’ reading and spelling abilities at the same time through innovative, interactive design and combine listening and speaking into German instruction with AR sound function. Choosing the right method for studying German may facilitate the learning process more effectively. This can help students improve a variety of learning motives, such as ‘care of the self’ or ‘the use of pleasure’ (Foucault 1984), or other factors formed by the blended action of survival needs and self-selection (Hennig 2013). AR picture books used in German instruction may drive learners’ language acquisition processes to a positive side through the accumulation of learning motivation and the innovation of pedagogical content.

## 3. Prestudy Comparison of German Teaching Effects between AR Picture Books and Traditional Picture Books

### 3.1. Research Background

Students’ satisfaction with picture books needs to be controlled due to the interference that may occur in the model (Kamphuis 1991; Woody et al. 2010). It is usually associated with both cognitive and emotional aspects (Oliver 2014). Cognition implies that when the reward is higher than the expected value, satisfaction will be generated; otherwise, dissatisfaction will be produced (Oliver 1980). Emotions, another important component of satisfaction (Phillips and Baumgartner 2002), play a significant role (Ladhari 2007). They describe the mood of the moment and help one evaluate how pleasant it is to feel something (Paulssen and Birk 2007). Therefore, the assessment of satisfaction with picture books usually takes into account students’ cognition of the gap between their expectations and reality (Szymanski and Henard 2001), as well as how they feel after reading picture books (Mai and Ness 1999). These two aspects jointly explain satisfaction with picture books (Bigné et al. 2005).

### 3.2. Research Purpose

For the prestudy, we collected picture book samples through the internet and library, and we classified and selected two representative picture book samples with high and low satisfaction levels to avoid possible influences caused by different satisfaction levels in Studies 1–3.

### 3.3. Methods and Materials

The respondents were college students, including students majoring in German, students who chose German courses from different majors, or students who took German courses outside of school. We did not choose children’s picture books; that is, we selected the picture book sample for an audience whose scope was close to the target group of young people and adults (Kümmerling-Meibauer 2015). We (the nine authors of the present study) discussed and decided whether the collected picture books met this standard at an internal meeting. The criteria included whether the students were interested, willing to try reading, and willing to keep reading. Some picture books involve children’s stories, but many college students enjoy them, so we included them in the collection scope. In total, we selected 30 samples of picture books. To eliminate possible interference from the picture book samples—such as readers’ potential preference and selection of picture books (Robinson et al. 1997), or the interaction effect caused by the combination of image narratives and text narratives (Wesseling 2004)—we performed multivariate scale and cluster analyses to cluster the samples. Each picture book was given to the subjects, who were asked to categorise them into groups. The classification was based on the subjects’ belief that the samples in each group had high similarity, and little difference in preferences for samples in the group after classification. The number of groups and the number of samples within each group were not restricted. At the end of the clustering process, representative samples from each group were chosen according to our internal meetings. The selection was based on similarity with other samples in each respective group.

Picture books were classified from September to October 2020. The respondents were 60 college students (28 males and 32 females). There were 13 freshmen (21.7%), 17 sophomores (28.3%), 15 juniors (25.0%), and 15 seniors (25.0%). After gathering the classification results, we converted the outcomes of graph card clustering from the correlation frequency matrix to a different frequency matrix, and we further calculated the Euclidean distance between each sample and the corresponding six-dimensional coordinates using multivariate scale analysis.

At the end of grouping, we tested students’ satisfaction with the representative samples of each group. We conducted the study in November 2020, and 68 college students participated in the evaluation (28 males and 40 females). There were 17 freshmen (25.0%), 16 sophomores (23.5%), 23 juniors (33.8%), and 12 seniors (17.6%). The questionnaire used for the test was the 3-question satisfaction scale proposed by Rouibah and Al-Hassan (2019). We performed ANOVA on the picture book samples with the highest and lowest satisfaction levels to establish whether there were significant differences in students’ satisfaction with the two samples. The chosen picture books with the highest and lowest satisfaction levels were redrawn as Chinese–German bilingual picture books, and AR interaction points were added at text markers to achieve the AR effect (Tang 2021).

### 3.4. Results

We carried out multivariate scale analysis to obtain the coordinates of each sample in six-dimensional space. The fitting results showed that Kruskal’s stress value was 0.0673 < 0.10 and Young’s S-stress value was 0.080 < 0.10, both within the acceptable standard. RSQ = 0.963 indicated that the explanatory power of the model reached 96.3% and that the data fit well (Kruskal 1978).

**Figure 2:**
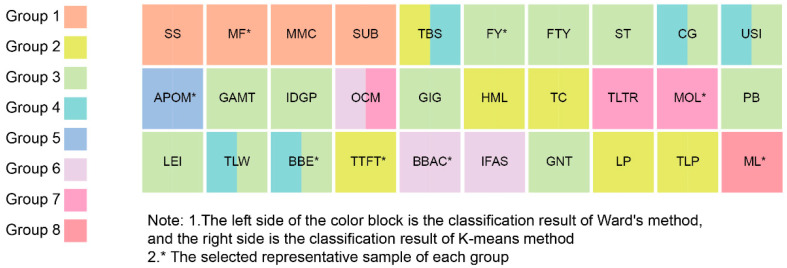
Classification outcomes of the picture book samples and the selection results of the representative samples.

Furthermore, we used hierarchical cluster analysis to compute the six-dimensional data via Ward’s method without specifying the number of clusters. The results indicate that in the last ten clustering cycles, the incremental percentage of some agglomeration coefficients has increased. The next-to-last shot rose from 30.79% to 102.59%, the fourth-to-last shot from 31.08% to 42.02%, the fifth-to-last shot from 18.81% to 31.08%, and the eighth-to-last shot from 19.54% to 24.19%. This signals that clustering with these four cluster numbers may have been more reasonable. Therefore, we repeated hierarchical cluster analysis using the method of specifying the number of clusters. The MANOVA results of the post-clustering samples showed that only the eight-group clustering exhibited significant differences in all six dimensions (*p* < 0.05), implying that the eight-group clustering had the least number of groups and the cleanest intergroup segmentation among all clustering. In addition, we employed Cohen’s kappa test to compare the grouping outcomes of the eight groups specified by Ward’s method with those of the eight groups specified by the k-means method; κ > 0.5 and *p* < 0.05, indicating no significant difference between the two groups (Chen et al. 2010). We verified the accuracy of hierarchical cluster analysis. Figure 2 presents the classification outcomes of each picture book sample and the selection results of the representative samples. For copyright reasons, the samples in this paper are denoted by code name.

Satisfaction tests on a representative sample of picture books demonstrated that the BBAC had the highest satisfaction among the eight representative samples; AVG = 4.201, SD = 0.724. Satisfaction with FY was the lowest; AVG = 3.843, SD = 0.772. The ANOVA of the two samples showed that the statistics of Levene’s test were not significant; W = 0.789, *p* = 0.376. It meets the homogeneity hypothesis. As indicated in Table 1, there were significant differences between the sample groups; F = 7.776, *p* = 0.006. There were differences in satisfaction between the two groups, so these two picture books were suitable for selection as representative samples.

**Figure 3:**
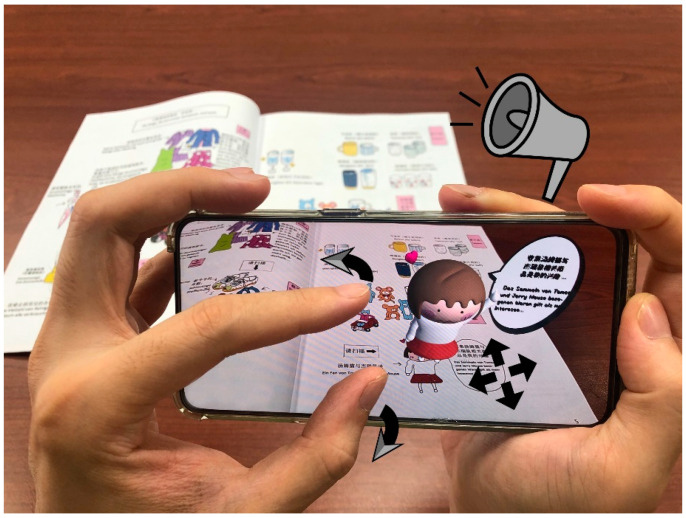
Bilingual AR picture book sample.

We produced the AR sample through Unity Vuforia (Santoso and Gook 2012), the most commonly used AR development platform in the world; over 30,000 apps have been created with Vuforia in the AppStore and Google Play (Barrile et al. 2018). Vuforia utilises a database containing user-defined object information, which is employed by the camera to compare the content in the viewfinder in real time, obtain 3D coordinates when the object appears, and display predefined virtual content on a realistic basis in a tracking manner. The interactions with AR picture books in this study include three aspects. The sample production outcomes of the picture book are shown in Figure 3. The first aspect entails scanning and model presentation. Students can use phones equipped with software to scan objects and study with virtual objects that appear in books. For students, the model presentation adds the dimension of a picture book, and the process of students scanning and the models appearing is an interaction (Chen and Wang 2015). Interactive learning environments can have positive effects on learning (Ho et al. 2017). Therefore, AR technology may help with German instruction.

The second aspect is that students control the model’s size through gestures after scanning the interaction effect. The programme obtains the 2D coordinate points behind the first touch screen of the two fingers and the corresponding new 2D coordinate points after real-time movement, by comparing the difference value of the coordinate points to judge whether the current gesture implies enlarging or shrinking.

The third aspect indicates the use of AR picture books to achieve audio reading. The text of each AR interaction is pre-recorded by voice. The sound continues to play when the target image is continuously tracked; conversely, the sound is turned off if the target image is lost.

### 3.5. Discussion

The main purpose of this study was to determine the suitability and match of the tested object and sample, and to eliminate external factors that may interfere as much as possible. Due to the complexity of language picture books and the uncertainty of personal perception, the importance and contribution of this step are self-evident. At the same time, the screening and testing steps in this phase can be used as a reference for subsequent research. When faced with complex samples, screening can be carried out through the above steps to address a variety of research topics. In addition, at this stage, through the classification and screening of picture book samples, the cost of subsequent AR sample production is reduced, the efficiency of the overall study is improved, and the foundation for subsequent research is established.

## 4. Study 1: Students’ Learning Interest: A Comparison between AR Picture Books and Traditional Picture Books

### 4.1. Research Background

AR technology can contribute to the presentation and interpretation of text in an interactive manner (Dünser and Hornecker 2007b). Compared with the marks of traditional picture books, AR picture books can help readers recall the story (Dünser et al. 2012). Because of the added AR elements, readers may be more willing to understand the content presented in the text. AR picture books can make text more lively due to the visualisation of abstract concepts, so they play a crucial role in learning (Zainuddin et al. 2009). Previous research has proposed a system for shaping a new mixed reality reading experience, creating entertaining, educational books that not only provide visual stimulation, but which also contain additional interactive properties. Using AR for text reading adds extra interactive attributes as well as traditional methods (Clark and Dünser 2012). AR technology in foreign language learning can improve students’ performance by making their experience more immersive and inspiring. Students have an incentive to use AR picture books, and AR learning can enable them to study more actively (Mahadzir and Phung 2013). We developed a mobile AR foreign language learning app to create a more interesting, efficient, and interactive learning experience. Through the test, we found that students have a high degree of recognition for the ease of use and practicability of mobile AR English learning apps, and AR foreign language learning provides novel, interesting techniques to enhance one’s willingness to learn. Most students can accept the AR learning environment and improve their intent to participate in learning (Hsieh et al. 2014). Two years of observation and the evaluation of three learning groups showed that, with feedback from open-ended focus group interviews and self-reflective papers, using e-learning and face-to-face learning are complementary. Interactive media can complement the spontaneity and motivation achieved face-to-face in the classroom (Donnelly 2010). Similarly, using e-learning, López-Pérez et al. (2011) pointed out in the teaching effectiveness evaluation that interactivity increases learning motivation in the learning process. AR technology may attract students and help them become more driven to learn due to its interactivity and innovation, but it could also cause confusion in learning given the possible conflation of virtual and real integration with excessive autonomy (Dünser and Hornecker 2007a; Kennewell et al. 2008). Hence, it is necessary to determine whether AR picture books can bring positive benefits to university-level German instruction.

### 4.2. Research Purpose

The study compared AR picture books (AR technology for text markers) with traditional books (underlined and bold for text markers) to explore whether AR picture books could help students significantly improve their learning interest in German courses in college rather than traditional books.

### 4.3. Methods and Materials

In Study 1, we divided the students into the experimental group using AR picture books and the control group using traditional picture books in order to compare learning interests. Notably, satisfaction with picture books may be a variable that affects the results (Kurucay and Inan 2017; Lin and Chen 2016). We controlled for equal numbers of students in the two groups based on the study design. Half of the students in each group employed the high-satisfaction picture book, while the other half utilised the low-satisfaction picture book. All students were German majors. For 20 min, the students were allowed to study freely through picture book samples corresponding to the group to which they belonged. The learning interest questionnaire used is from the 11 questions by Hwang and Chang (2011). After receiving the questionnaire, we performed ANOVA difference analysis on the results in the traditional picture book group and the results in the AR picture book group to test whether AR text marking can help to improve students’ interest in German learning (versus traditional marking).

We conducted the study from December 2020 to January 2021. A total of 120 college students (39 males and 81 females) participated in the evaluation after reading picture books in the study. There were 23 freshmen (19.2%), 33 sophomores (27.5%), 28 juniors (23.3%), and 36 seniors (30.0%). We assigned 60 students each to the traditional picture book group and the AR picture book group, respectively. In each group, 30 students read the high-satisfaction picture book (BBAC), while the other 30 students read the low-satisfaction picture book (FY).

### 4.4. Results

The results showed that the statistics of Levene’s test were not significant, W = 0.188, *p* = 0.665. It meets the variance homogeneity hypothesis. As shown in Table 2, students in the traditional group had a relatively low learning interest in German, AVG = 3.073, SD = 0.539. Students in the AR group had higher learning interest, AVG = 3.306, SD = 0.554. There were significant differences between groups, F = 5.470, *p* = 0.021. Compared with traditional picture books, students use AR picture books to learn German with a higher learning interest.

### 4.5. Discussion

In German learning, by comparing students’ interests in using AR picture books with those of traditional picture books, AR picture books show a better role in promoting teaching than traditional picture books. Students use AR picture books to learn German, resulting in a higher learning interest. The research results echo previous research showing that AR flashcards can help Taiwanese students strengthen their learning interest in foreign language learning (Tsai 2018). On the whole, it also conforms to the literature that using AR technology in classroom teaching can help improve learning efficiency (Yip et al. 2019). Therefore, to obtain a better teaching effect in German teaching at the university level, using AR picture books as textbooks is an effective method.

## 5. Study 2 The Teaching Effect of AR Picture Books Applied to German Learning

### 5.1. Research Background

In the process of education through digital books, satisfaction is a factor that is often discussed (Wang et al. 2019). A study on the application of a 3D virtual environment in games to language teaching shows that there is a close relationship between students’ satisfaction with games and their learning motivation (Lan et al. 2018). Previous studies have shown that satisfaction is a determinant of students’ willingness to continue learning when blogs are used as learning tools to assist college students (Ifinedo 2018). Students’ satisfaction with AR picture books may also be one of the factors affecting their willingness to learn German. In this paper, satisfaction with AR Picture Books was used as a classification variable for intergroup comparison. In addition, for learners, German proficiency is obviously different. Some students may just start to learn and still have a poor German proficiency, while the other students have a certain grasp of German. Strong readers were able to recall more information from books than weak readers, but there was no difference when they read AR picture books (Dünser 2008). This suggests that AR picture books may help bridge the ability gap between readers. However, some people think that the benefits of text marking for learners of low-level language proficiency may not be as obvious as those of high-level learners because readers cannot make use of them effectively (Degand and Sanders 2002). Therefore, this study differentiates the German proficiency of students. In addition to controlling for the possible influence of variables, it can also test whether AR picture books can bring different help to students with different German proficiencies. The German proficiency of students is established as another factor to be classified.

### 5.2. Research Purpose

The study examines the effects of high vs. low satisfaction in AR picture books and learners’ German proficiency level (high vs. low) on the following three student outcome variables: (1) learning interest, (2) continuous learning intention, and (3) learning results.

### 5.3. Methods and Materials

Differences between groups were examined using 2-way MANOVA. The factor of students’ satisfaction with AR picture books is a binary category variable. We controlled for the reading of high-satisfaction picture books or low-satisfaction picture books. We classified the other factor, German proficiency, according to the pre-inquiry and judgement of the students’ German proficiency before the study. They are divided into the following two categories: better and poor German proficiency. Thus, students were divided into 4 groups in a 2 × 2 design. German proficiency was determined by asking the students to give a 1-min self-introduction. Next, they were asked about their self-introduction in a chat format and to give a short answer. We decided on the students’ German proficiency at an internal meeting according to the students’ self-introduction and answers. They had to study the AR picture books of their group independently for 20 min and then fill in the corresponding questionnaire after learning. In the study, we used the 11 questions for learning interest proposed by Hwang and Chang (2011). Watanabe et al. (2011) proposed 7 questions for continuous learning intention. The four questions for learning outcomes were proposed by Zhan and Chen (2011). A total of 22 questions tested students’ perceptions and preferences for using AR picture books to learn German.

The study took place in January 2021. A total of 114 undergraduate students (59 men and 55 women) were invited to participate in the assessment. There were 26 freshmen (22.8%), 24 sophomores (21.1%), 29 juniors (25.4%), and 35 seniors (30.7%). There were 30 students who watched high-satisfaction picture books (BBACs) with better German proficiency, 25 students who watched high-satisfaction picture books (BBACs) with poor German proficiency, 29 students who watched low-satisfaction picture books (FYs) with better German proficiency, and 30 students who watched low-satisfaction picture books (FYs) with poor German proficiency. Based on estimates using G*Power3.1, the effect size F2 was 0.25, the α err prob was 0.05, the power was 0.95, and 36 total sample sizes were needed to test the total effect, thus meeting the sample size premise (Faul et al. 2009).

### 5.4. Results

The equality test results of the covariance matrix show that Box M = 29.315, F = 1.547, and *p* = 0.064, thereby meeting the analytical premise. The statistics of Levene’s test were not significant for all constructs. For the construct of learning interest, W = 0.980, *p* = 0.405; for the construct of continuous learning intention, W = 1.599, *p* = 0.194; and for the construct of learning outcomes, W = 0.192, *p* = 0.902. Hence, the variance homogeneity hypothesis was supported. The two-factor interaction was not significant, F = 0.981, *p* = 0.405. This indicates that satisfaction and German proficiency did not influence each other in terms of the perception results. The findings of the MANOVA test are shown in Table 3. Satisfaction code 1 represents the high-satisfaction picture book (BBAC), and code 2 denotes the low-satisfaction picture book (FY). As for German proficiency, code 1 indicates better proficiency, and code 2 signals poor proficiency. The results demonstrate that regardless of whether the students had good or poor German proficiency, their learning interests, continuous learning intentions, and learning outcomes were significantly higher after reading picture books with higher satisfaction. This suggests that adding satisfaction can effectively help students gain a more positive perception in the process of learning German.

On the other hand, compared with students with poor German proficiency, students with better German proficiency had significantly higher perceptual results in all constructs. The findings are presented in Table 4. This further validates the view proposed by Noels et al. (2016) that language learning is a process of approaching two poles; positive perception and proficiency may continuously boost learning and enter an ongoing positive cycle. In contrast, entering a negative cycle may lead to a decrease in one’s sense of achievement and further reduce learning efficiency.

### 5.5. Discussion

The findings indicate that using AR picture books with higher satisfaction in German instruction can help students obtain a higher learning interest, continuous learning intentions, and learning outcomes. Students’ satisfaction with picture books is a crucial factor in impacting the teaching effect. The results echo previous studies showing that improving students’ satisfaction with teaching aids is conducive to better teaching results (Lan et al. 2018). To obtain higher satisfaction with picture books, the design and production of AR picture books is critical. Second, the findings imply that students with higher levels of German proficiency will experience higher learning interest, continuous learning intention, and learning outcomes when using AR picture books. The results again verify previous research that found students with higher language proficiency can effectively use the help brought about by text marking (Degand and Sanders 2002). The assistance engendered by AR picture books in German instruction will be enhanced along with the improvement of students’ proficiency.

## 6. Study 3: Model Construction to Improve Satisfaction

### 6.1. Research Background

It is vital to examine how to improve students’ satisfaction with AR picture books. Bilingual picture books are helpful in boosting language ability (Agosto 1997; Martens et al. 2012). The learning process may be related to the narrative and integrity of picture books, and interaction may play a certain role after the introduction of AR. Interactivity enables students to interact with picture books while learning, permitting them to experience sensory stimulation, which may enhance the learning process (Dias 2009). By investigating the influence of design elements that need to be considered in the design of picture books (such as narratives, integrity, and interactivity) on students’ satisfaction with AR picture books, a model is established to facilitate German proficiency. In addition, the model, combined with reference to the existing literature, reveals that peer interaction may have an impact on learning-related perceptions (Philp et al. 2013), and in the learning process, peer relationships may play an important role as a moderating variable in the model. Based on the exploration of the relationship model, it helps to think about the design of AR picture books from the perspective of interaction design and to consider suggestions for innovative approaches to German instruction.

### 6.2. Research Purpose

The study established a structural equation model and proposed design suggestions for AR picture books. The influence relationship and path coefficient among the following five variables are further addressed in the model: (1) perceived interactivity, (2) perceived narrativity, (3) perceived integrity, (4) flow experience, and (5) satisfaction. At the same time, we tested whether the path relationship was affected by the adjustment effect of peer learning.

### 6.3. Research Hypothesis

**Figure 4:**
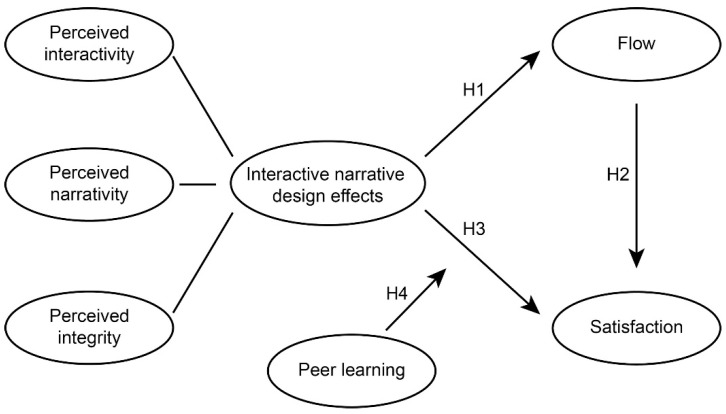
Research hypothesis diagram.

The research hypothesis is shown in Figure 4. The process of presenting content in a picture book after adding AR elements is an interactive narrative (Nam 2015). Users can give instructions to the storyline based on their actions (Riedl and Bulitko 2013). Developing high-quality interactive experiences is an artistic process (El-Nasr 2007). Some researchers are already thinking about interactive narratives’ effectiveness in education, advertising, reasoning, and other applications. It shares similarities with the problem-based approach of explorative learning, which helps students solve a series of interrelated challenges (Rowe et al. 2011). In AR picture books, users are limited to the role of observers, and the influence of participation on narratives can be secondary (Ryan 2008). Weak interaction allows for more opportunities for predesigned, clever ideas to be displayed (Crawford 2012). An interactive narrative depends on how users participate, the structure of the interactive nodes, and whether the story is a complete one (Ryan 2006). These characteristics correspond to perceived interactivity, perceived narrativity, and perceived integrity in user psychology. We will discuss the influence of INDE in the model.

The experience of participating in the story is positively correlated with the perception of flow (Szilas and Ilea 2014). The interaction process and flow have a direct and influential relationship (Kim et al. 2005). When AR picture books are applied in German instruction, students’ recognition of INDE may also provide positive effects on flow. Therefore, we proposed the following:

**Hypothesis 1** **(H1).** *INDE has a positive impact on flow*.

In the study of user behaviour, flow is often regarded as the centre, connecting the experience structure and influencing the final perception (Siekpe 2005). Flow and satisfaction have a positive relationship with each other in many fields, including information technology (Xin Ding et al. 2010) and information systems, according to Deng et al. (2010). Flow and satisfaction may be equally closely related in German instruction. Hence, we posited the following:

**Hypothesis 2** **(H2).** *Flow has a positive impact on satisfaction*.

The quality of human-computer interaction affects user satisfaction (Xin Ding et al. 2010). The better the interaction design, the better the satisfaction, and the design of a reasonable interaction process is one of the methods to improve satisfaction (Yannakakis 2008). Many games experiment with a variety of interactive narratives to increase satisfaction, as games can achieve player satisfaction through the design of interactive narratives (Yannakakis and Hallam 2009; Young and Cardona-Rivera 2011). INDE and satisfaction may have the same influential relationship in the process of German teaching. As such, we developed the following:

**Hypothesis** **3** **(H3).** *INDE has a positive effect on satisfaction*.

Peer learning and satisfaction are often discussed together. A study of university students in Malaysia revealed that peer learning made students more satisfied with the learning process (Lim et al. 2020). Peer learning also has a linear relationship with satisfaction in the graduate education stage (Lee et al. 2021). However, it seems more reasonable for two constructs to be set as moderators in this model. From the perspective of the effect of AR picture books on German instruction, it is undoubtedly of great significance if the type of learning state can amplify the influence of INDE on satisfaction. As such, we came up with

**Hypothesis** **4** **(H4).** *Peer learning has a positive, moderating effect on the relationship path between INDE and satisfaction*.

### 6.4. Methods and Materials

For Study 3 we used a structural equation model to test the relationship between the models of INDE, flow, and satisfaction. The AR German picture book employed in the study is a high-satisfaction picture book (BBAC). We recruited participants in two ways. Students in the peer learning (PL) group were asked to invite close friends to form small groups to participate together. Students in the independent learning (IL) group participated alone. A total of 286 students from the PL group and 228 from the IL group were invited. We conducted the study from April to May 2021. A total of 514 college students participated (230 males and 284 females). There were 124 freshmen (24.1%), 117 sophomores (22.8%), 151 juniors (29.4%), and 122 seniors (23.7%).

The questionnaire included 7 questions from Chen (2010) and Lin and Chang (2018) to measure perceived interactivity. Yang et al. (2010) proposed 5 questions to gauge perceived narrativity, as well as Lin et al. (2019). Sun et al. (2017) proposed 4 questions to measure perceived integrity. In addition, the questionnaire included 10 questions from Rheinberg et al. (2003) to measure flow, Rouibah and Al-Hassan (2019) proposed 3 questions to gauge satisfaction.

### 6.5. Results

#### 6.5.1. Hypothesis Test of Data Distribution

In the PIN, Cronbach’s α was greater than the current reliability after deleting item PIN1. PIN2, PIN5, and the commonality of Pin6 was less than 0.50. In the PN, the commonality of Pn4 and Pn5 was less than 0.50. In the PIG, the commonality of PIG4 was less than 0.50. In the FL, the commonality of FL1, FL2, FL3, FL6, FL8, FL9, and FL10 was less than 0.50. Hence, we deleted the above item.

#### 6.5.2. Reliability Analysis

The reliability analysis results are depicted in Table 5. The Cronbach’s α of each construct was greater than 0.6, and the new reliability when any item was deleted was lower than the current reliability (Hatcher and Stepanski 1994; Morgan et al. 2004). The corrected item-total correlation coefficient for each item was greater than 0.3 (Sakip et al. 2015). In sum, the representation had good construct reliability.

#### 6.5.3. Exploratory Factor Analysis

The results of exploratory factor analysis are displayed in Table 6. The KMO value of each construct was greater than 0.60, indicating that the sum of the square of simple correlation coefficients between the items was greater than the sum of the square of partial correlation coefficients (Garcia-Maraver et al. 2015). Bartlett’s sphere test (*p* < 0.05) indicates that the correlation coefficient matrix of the item was not a unit matrix, which is suitable for factor analysis (Bartlett 1937). On the other hand, the commonality of each item was greater than 0.50, showing appropriate commonality (Alvarenga et al. 2012). The factor load was greater than 0.50, which is higher than the medium level (Shevlin and Miles 1998). Total variance explained exceeded 50% (Tahar et al. 2010). The test results show that items did not need to be deleted further. In factor analysis, only one new factor with an eigenvalue greater than 1 can be extracted from each construct, which can be verified by a single-construct test (Kohli et al. 1998).

#### 6.5.4. First-Order Confirmatory Factor Analysis

**Figure 5:**
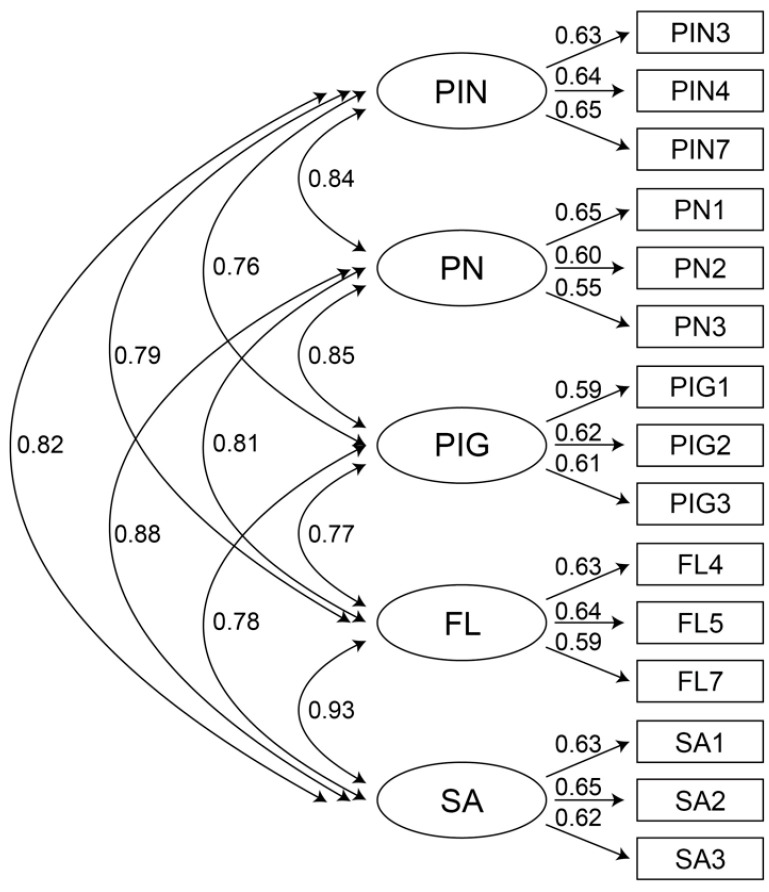
First-order CFA model.

As shown in Figure 5, all potential variables were correlated, satisfying the premise of path analysis. In addition, all the fitting indices of the model met the recommended standards, as seen in Table 7, indicating that the first-order confirmatory factor analysis (CFA) model had a good fit (Fornell and Larcker 1981).

The results of convergent validity are presented in Table 8. In the first-order CFA model, the factor load of each item was greater than 0.5. The ratio of the coefficient estimate to the standard error was significant, *t* > 1.96, *p* < 0.05, which met the fitting index. The combined reliability (CR) of each construct was greater than 0.6 (Rong-Da Liang and Lim 2011), and the average variance extracted (AVE) was greater than the base value of 0.36 (Fernandes et al. 2018). In sum, the items had convergence validity.

The AVE method was used to evaluate the discriminant validity between constructs. The results of the discriminant validity test are depicted in Table 9. The square root of the mean variance extracted from each construct was greater than the Pearson correlation coefficient between each construct and the other constructs, indicating that the item had discriminant validity (Fornell and Larcker 1981).

#### 6.5.5. Second-Order CFA

**Figure 6:**
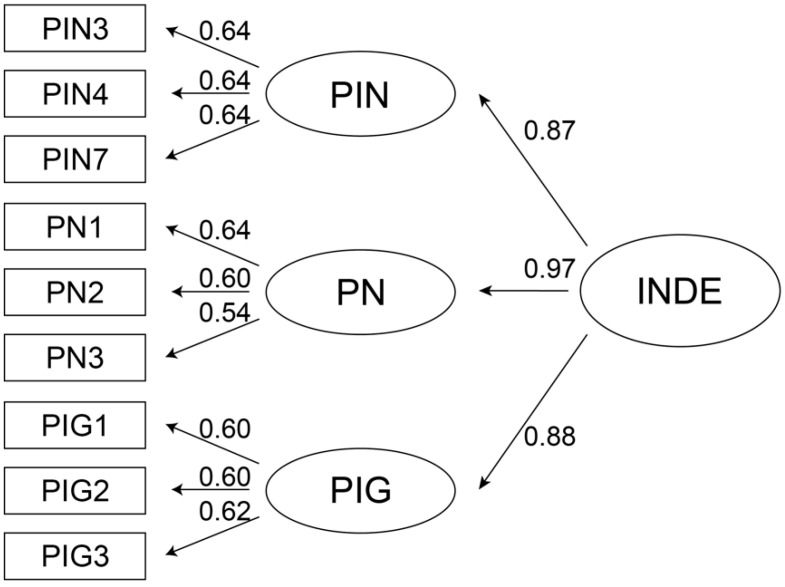
Second-order CFA model.

Based on the Ryan (2008) explanation the of interactive narrative, it comes from users’ perception of interactive products from the following three aspects: interactivity, the narrative, and integrity. Theoretically, it is possible to extract the second-order INDE from the three constructs. The results of first-order CFA revealed a high correlation between the constructs, so it was suitable to use oblique second-order CFA to test the model relationship. As presented in Figure 6, there was a large coefficient between INDE, perceived interactivity, perceived narrativity, and perceived integrity.

The model fitting indices are displayed in Table 10, and all the model fitting indices meet the recommended standards. CFI = 1 and RMSEA = 0, indicating a very good fitting outcome (Baik et al. 2020). This suggests that the second-order CFA model had a better model fit. Hence, INDE was suitable to be used as the second-order construct of perceived interactivity, perceived narrativity, and perceived integrity to participate in model construction.

#### 6.5.6. Results of the Structural Equation Model

**Figure 7:**
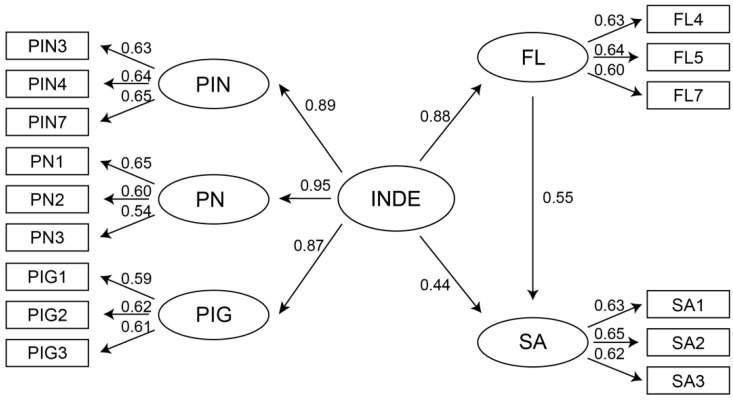
Structural equation model.

In the process of learning German by using AR picture books, INDE had positive path coefficients for flow and satisfaction. The model path relationship is portrayed in Figure 7. The model fitting indices are shown in Table 11, all of which were higher than the recommended standard values. The model also had a very good fitting index with CFI = 1 and RMSEA = 0, indicating that the model was constructed reasonably and achieved the standard fit degree (Fornell and Larcker 1981).

The test results of the direct and indirect effects of the path are shown in Table 12. First, the direct influence of INDE on the flow path coefficient (β = 0.876) and the two-tailed significance (0.001) were estimated using the bias-corrected percentile method. INDE had a positive, direct, and significant impact on flow. This indicates a positive causal relationship between the students’ perception of INDE and the flow they experienced when using AR picture books to learn German. Therefore, H1 was supported, and the second-order construct INDE had a positive influence on flow.

Second, the direct influence of flow on the satisfaction path coefficient (β = 0.553) and the two-tailed significance (0.006) were estimated using the bias-corrected percentile method. This reveals that when students used AR picture books to learn German, the stronger the flow they felt, the higher their satisfaction with the picture book. There was a direct, significant positive causal relationship between the two. When designing AR picture books for German instruction, more consideration should be given to how to improve students’ flow in the process of use, which is conducive to enhancing satisfaction with picture books. Hence, H2 was supported, and flow had a positive influence on satisfaction.

Finally, the direct influence of INDE on the satisfaction path coefficient (β = 0.436) and the two-tailed significance (0.095) was estimated using the bias-corrected percentile method. The path coefficients between constructs were not significant. Although students had a direct influence on INDE and satisfaction with AR picture books, this effect was relatively weak and not statistically significant. Although there was no direct causal relationship between students’ perception of AR picture books, INDE, and satisfaction, based on the intermediary role played by flow between them, we can assume that INDE and satisfaction had a significant, indirect influence through the intermediary of flow. The indirect influence coefficient (β = 0.484) and the two-tailed significance (0.005) were estimated by the bias-corrected percentile method. Because there was no direct path relationship between INDE and satisfaction, flow, as an intermediary variable, involved complete mediation. In general, under the mediating effect of flow, the path coefficient of the total influence of INDE on satisfaction was β = 0.920, and the two-tailed significance (0.001) was estimated using the bias-corrected percentile method. Thus, the better INDE helps to improve satisfaction when AR picture books are used to learn German. Hence, H3 was supported, and the second-order construct, INDE, had a positive influence on satisfaction.

The state categories of students included the peer learning (PL) group and the independent learning (IL) group. The path coefficients, PLβ and Ilβ, between INDE and satisfaction in the data models of the two groups are labelled. A new competition Model A was added, and the nihility hypothesis (PLβ = Ilβ) was established. Competition Model A was compared with the initial model to test the moderating effect. As shown in Table 13, there was no significant difference between competitive Model A and the initial model (*p* > 0.05). The nihilistic hypothesis (PLβ = Ilβ) was accepted. For the path relationship between INDE and satisfaction, peer learning or independent learning did not produce a significant moderating effect. This means that in the process of learning German by using AR picture books, whether students were in the peer learning or independent learning groups, this would not affect the path of INDE to satisfaction. Therefore, H4 was not supported, and in the relationship path between INDE and satisfaction, peer learning produced a positive moderating effect that was rejected.

### 6.6. Discussion

As an interactive teaching aide, AR picture books are affected by INDE when presenting text content. This influential process is achieved by changing students’ flow experiences. The importance of flow in teaching has been verified again (Giasiranis and Sofos 2017). INDE is successfully defined as perceived interactivity, perceived narrativity, and perceived integrity in this model. This indicates that, to be more conducive to German teaching, when creating AR picture books, designers should give full consideration to how students participate in the interaction, the text presentation method, and the complete presentation of text content. Importantly, peer learning does not promote the learning effect as effectively as expected. This may be because the interaction of AR picture books requires students to cooperate with the presentation of text content, which requires an extra cognitive load (Radu 2014). Frequent discussions lead to students’ inability to focus on ongoing AR learning and break away from the teaching context provided by AR picture books.

## 7. General Discussion

In recent years, AR technology has played a vital role in many university courses due to its innovation in teaching methods (Yuen et al. 2011). The exploration of AR applications in German education is a critical part of educational thinking and improvement. Through one prestudy and three studies, we explored the application of AR picture books in German instruction, as shown in Table 14. For the prestudy, we used cluster analysis and ANOVA to select two representative samples of picture books with high and low satisfaction, thus paving the way for subsequent studies. For Study 1, to prove that AR picture books can assist with teaching, we used ANOVA to compare the use of AR picture books in German instruction to increase students’ learning interest (versus traditional picture books). For Study 2, to prove the influence of the two variables of AR picture textbook satisfaction and students’ German proficiency, we performed a 2-way MANOVA to show that drawing ability with higher satisfaction would increase learning interest, continuous learning intention, and learning outcomes. At the same time, Study 2 indicates that students with higher German proficiency had greater learning interest, continuous learning intention, and learning outcomes. According to the results of Study 2, improving students’ satisfaction with AR picture books in German instruction is key to obtaining a more positive learning perception. To explore ways to effectively enhance satisfaction with AR picture books, we conducted Study 3 to establish a structural equation model to investigate the impact of INDE, flow, and peer learning on satisfaction. This paper provides some important findings through step-by-step verification, which we will discuss in detail.

The results confirm that, compared with traditional picture books, AR technology can more strongly promote students’ interest in learning German. The use of AR technology for marking picture books is very inclusive. There is no negative or abrupt combination between AR technology and picture books, and the use of interaction design as a teaching aid shows its potential. Because AR picture books can boost students’ interest in learning German, teachers can try to use AR picture books to teach when they believe that students need encouragement to promote the positive cycle of the learning state. For students in a positive mental state of learning, AR picture books may help them to sustainably maintain their enthusiasm for learning. For students in a negative psychological state of learning, AR picture books may help to improve current laziness or resistance and return them to a virtuous circle of learning.

We comprehensively evaluated students’ learning states in the process of studying German based on the three constructs of learning interest, continuous learning intention, and learning outcomes. What is exciting is that in the two groups with better German proficiency and poor German proficiency, the satisfaction of students with AR picture books shows a significant influence on their learning state. The improvement of satisfaction can effectively help students obtain higher learning interest, continuous learning intention, and learning outcomes to establish a good learning state. This indicates that the design research of AR picture books is important when they are used in German instruction. An excellent AR picture book design increases students’ satisfaction and plays a better role in teaching. As a crucial construct of evaluation teaching tools, satisfaction plays a substantial role in the process of using AR picture books in German instruction. Hence, it is vital to conduct in-depth design research on AR picture books so that students can be more satisfied with them.

On the other hand, in the group with high satisfaction and the group with low satisfaction, as a dividing construct, students’ German proficiency had a positive effect on learning interest, continuous learning intention, and learning outcomes. Students with better German proficiency had higher learning status than those with poor German proficiency, which points to the continuous effect of learning psychological polarisation in German learning. When students gain a feeling of small achievement, they can continue to accumulate a positive learning state. On the other hand, students with poor German proficiency may have corresponding resistance due to constant self-denial or fear of unknown learning content. There was no significant interaction between satisfaction and German proficiency. This implies that we cannot quickly adjust students’ German proficiency through the design of AR picture books. Language learning is a cumulative process, and students can only improve their ability from poor to good through long-term ongoing learning. It is important to encourage and actively guide students in teaching. Giving encouragement to students can promote their learning psychology in a positive cycle and help them accumulate knowledge points to enhance their German proficiency.

As an interactive design product, the AR picture book has the attribute of INDE when presenting the text of German pedagogical materials. The results verify that INDE comes from perceived interactivity, perceived narrativity, and perceived integrity. As Ryan (2006) said, the interactive way that users participate in interactive products, the division of interactive nodes in how interactive products present interactive text, and the completeness of presenting stories constitute the INDE of interaction design. This structure is also applicable in AR picture books. Through the structural equation model, INDE has a direct positive influence on flow and indirectly has a positive influence on AR picture book satisfaction through the mediator of flow. This indicates that sufficient attention should be given to perceived interactivity, perceived narrativity, and perceived integrity in the design of AR picture books for German teaching. Perceived interactivity requires AR picture books to interact with students in a reasonable way. For example, interactive experiences such as feedback and control, creation or cocreation, and self-adaptation can be achieved by using reasonable interactive behaviours, such as clicking, shaking, and sliding (Shedroff 1999). Perceived narrativity imposes requirements both on the content and presentation method of the text. The content of German instructional materials should be more narrative, and language learning should be fully combined with the story content, rather than simply combining grammar and text. In addition, in the process of combining text with interactive nodes in AR picture books, the re-disconnection and recombination of teaching text in a reasonable way should be based on the principle of retaining or improving the narrative nature of the original text. Perceived integrity is closely related to the full rendering of textual content. The design of AR picture books should reduce the possible pause (or even complete termination) of reading caused by adding the AR effect. The presentation of the story should be as comprehensive as possible. AR picture books should be designed on the premise of maintaining the integrity of the teaching text, and students should be given the right to read as completely as possible in instruction.

We also found that flow can directly influence satisfaction. Flow implies self-efficacy or the perception of challenge generated during interaction, which produces a positive experience of total engagement when the challenge is of moderate difficulty (Hamari et al. 2016). AR picture books used in German teaching should be designed to maintain appropriate interaction difficulty in the process of students’ experience to obtain a stronger flow experience. For example, students may be required to perform an operation within a limited time or to imitate an action and score the accuracy of the imitation. Through the pre-test of the publishing task in AR interaction, the operation requirements that are not too difficult but have some difficulty for students are set. At the same time, attention should be given to combining the key points of knowledge that need to be mastered in the process of the operation to achieve an effective learning state when using AR picture books for learning German.

The test outcomes of peer relationships in the process of German learning show that the change in learning relationships in the peer learning group or independent learning group did not have an effective moderating effect on the path of INDE to satisfaction. The teaching results of some subjects indicate that peer learning can obtain the educational effect more efficiently (Hilsdon 2014). The use of AR picture books for German learning relaxes the requirement for companionship, and one can also study well. Adopting AR picture books for German learning is undoubtedly more inclusive in terms of learning conditions. Students are free to study German at a higher level of efficiency without being constrained by interpersonal ties in learning relationships.

## 8. Theoretical Contribution

The contribution of this paper to theoretical development includes three aspects. On the first hand, by comparing the influence of AR picture books and traditional books on students’ learning interest in German, it is proved that students who use AR picture books have higher learning interest. From the perspective of theoretical research, this survey shows that the language teaching research about AR picture books is meaningful. Although AR picture books have a higher threshold to use, the better teaching effect it brings deserves more exploration and theoretical research by researchers. In addition to proving the value of previous studies, a broader study of AR picture books as teaching aids in a variety of courses or for students with a various characteristics proved to be meaningful. In addition, through the comparison of this study, it is preliminarily proved that it is necessary to further study the time and method of using AR picture books as German learning AIDS. Secondly, since language learning is a process of long-term memory and application, learning interest and continuous learning intention related to long-term behaviors are equally important in teaching activities in addition to current learning outcomes. In the studies on language teaching, researchers need to clarify whether AR picture books, as a new technology, can attract students and motivate them to engage in long-term language learning. Through the comparison of these three dimensions, this research proves the close relationship between students’ satisfaction with AR picture books and German learning from a theoretical perspective. It reveals the necessity of research on AR picture book design. Thirdly, this paper integrates previous studies on the effects of flow theory and interactive narrative design and develops and verifies an interactive narrative design for learning model (INDLM) for AR picture book design. Different from mature theoretical models such as TAM or UTAUT2, which are often discussed in human-computer interaction studies, INDLM pays more attention to human-computer interaction design for education. Although previously mature models have been applied and developed in many different studies, theoretical models developed specifically for specific purposes of use have an advantage in terms of explanatory power and validity over widely applicable theoretical models in this technological explosion era. Therefore, the study of interaction design specifically for education is equally important as a valuable research direction. The main contribution of this paper to model proposal and theoretical development lies in the development of INDLM and confirms its importance in AR picture book design. This study also lays a theoretical foundation for the subsequent application of the model and pedagogical behavior research. This study confirms the importance of perceived interactivity, perceived narrativity, perceived integrity and flow experience in human-computer interaction design applied to education. In addition, this study challenges the effectiveness of the peer learning method in the context of human-computer interaction.

## 9. Managerial Implications

The results of this research on AR German picture books can help improve students’ learning experiences. The results of this study have special significance for digital education, especially for German language courses at university level. At present, no quantitative evaluation has been performed in the field of the teaching effect of AR picture books in German education. AR picture books change students’ learning experiences by transforming the learning content from the text presentation or watching and listening process of audio-visual materials into an interactive process. According to the results of this study, if students use AR picture books as a learning tool in the process of learning German, they can obtain a higher learning interest than traditional picture books. In addition, peer learning in the process of learning German by using AR picture books has been proved to have no obvious benefits compared with students’ independent learning. The research results provide a basis for teachers and learners to use AR picture books as teaching aids in the process of language learning, and the necessity of social and public learning environment is no longer emphasized, which reduces the requirement of peer learning for students in the process of learning in order to obtain better learning results. The results indicate that interactive learning courses and traditional courses may differ in teaching methods and educational conclusions. Teachers should reconsider interactive learning in addition to their accumulated classroom teaching experience. In addition, students’ satisfaction with AR picture books is very important, which will directly affect students’ learning interest, continuous learning intention and learning outcomes. Therefore, in German learning courses, designers should try to design AR picture books with higher student satisfactory. In addition, another practical contribution of this study is that INDLM can be used as a reference for designers in designing AR picture books for the purpose of education. Designers should pay attention to the perceived interactivity, perceived narrativity and perceived integrity of students’ learning materials, which means the way of interaction between AR picture books and students, the story sequence told by the text, and whether the text can be fully learned within the course should be paid attention to. At present, although the technology of producing AR picture books is approaching maturity, the higher equipment cost, and the learning cost make it difficult for schools and teachers to make extensive attempts to apply new technologies before its proved effectiveness. Nowadays, many schools and teachers have explored the application of multimedia in education. From offline local software, to online web interaction, and then to intelligent technology to empower the system. The use of digital education technology expands educational activities from classroom to network space, and even influences external social culture from educational system.

## 10. Conclusions

We explored the teaching effects of AR picture books as applied to German instruction in university education. As a vehicle of presenting texts and marking key learning content, AR picture books can enable students to obtain a better learning experience and more positive learning tendencies. When students use AR picture books for learning, the more satisfied they are with the picture books, the more positive their learning state will be. This underscores the importance of designing and researching teaching tools and improving their quality in the process of language teaching. By using AR picture books, students can learn German more happily and enter a positive cycle of learning psychology. In the process of learning German, the INDE of AR picture books plays an important role in the satisfaction of picture books and the students’ ultimate learning state. In the design of interactive teaching tools, we should pay more attention to interactivity, narratives, and integrity. At the same time, the research reveals that learning German with AR picture books has no requirement for the formation of peer relationships during learning.

The main research limitation of this research is that the subjects are from China’s mainland and Taiwan region. The sample is representative of native Chinese students to some extent. Although the survey results have some explanatory power for the learning process of college students, students from other countries and cultures may not be fully represented due to the differences in their upbringing environment and mother tongue system, which needs to be further verified by future studies. It is important to further explore other constructs that influence student satisfaction in AR picture book design. It is necessary to pay attention to the iteration of the structural equation model after adding new design elements. Deeper design and improvement of AR picture books can play a better role in German learning under the agency of satisfaction. Previous studies have shown that gender differences seem to be a moderating variable in learning German (Okuniewski 2014). We did not test for that in this study, and more comparisons can be made in future research. In addition, the results of this study indicate that AR picture books may also be beneficial to students’ learning of other foreign languages, which requires further examination in the future. Furthermore, the application of AR picture books in teaching requires the support of electronic devices and applications, which undoubtedly increases the capital cost of education and the cost of users’ learning of teaching facilities. Future studies should further explore whether other more standard ways to enhance picture books could be used, and the effectiveness of these teaching aids should also be tested. Our conclusion proves the effectiveness of AR picture books in college German instruction and suggests ideas for college language teaching. Especially for teaching minority languages, the influence of AR technology on students’ interest and efficiency in language learning can be further tested. For example, learning dialects to continue and preserve traditional culture, the training of college students majoring in specific languages. From a longer-term perspective, the application of AR in the language field makes traditional teaching more interesting, which is of great significance to teaching. In the future, AR devices can be mass-produced and placed in schools, shopping malls, and training institutions to increase the interest of the general public, students, and children in learning different languages. The actual contribution of this research can be spread to other fields, such as the development of children’s AR devices to meet the enlightenment education needs of preschool children and quick on-the-job training for new employees. AR has very broad prospects in the future. The complete research system and experimental analysis results of this study can be used as a reference for future research, and AR technology can be applied in more fields.

## Figures and Tables

**Table 1:** Satisfaction differences among picture books.

Source	Type III Sum of Squares	df	Mean Square	F	Sig.	Partial Eta Squared	Observed Power ^b^
BBAC-FY	4.354 ^a^	1	4.354	7.776	0.006	0.055	0.791
Intercept	2200.066	1	2200.066	3929.501	0.000	0.967	1.000
Error	75.025	134	0.560				
Total	2279.444	136					
Corrected total	79.378	135					

^a^ R^2^ = 0.055 (Adjusted R^2^ = 0.048); ^b^ Computed using alpha = 0.05.

**Table 2:** Differences between groups in learning interest.

Source	Type III Sum of Squares	df	Mean Square	F	Sig.	Partial Eta Squared	Observed Power ^b^
Traditional -AR	1.633 ^a^	1	1.633	5.470	0.021	0.044	0.640
Intercept	1220.668	1	1220.668	4087.853	0.000	0.972	1.000
Error	35.236	118	0.299				
Total	1257.537	120					
Corrected Total	36.869	119					

^a^ R Squared = 0.044 (Adjusted R Squared = 0.036); ^b^ Computed using alpha = 0.05.

**Table 3:** MANOVA test for satisfaction.

Dependent Variable	German Proficiency	Satisfaction Mean (1)	Satisfaction Mean (2)	Mean Difference (1–2)	S.E.	Sig. ^b^
LI	1	4.367	3.984	0.382 *	0.156	0.016
	2	3.487	2.897	0.590 *	0.163	0.000
CLI	1	4.429	4.059	0.369 *	0.165	0.027
	2	3.406	2.652	0.753 *	0.171	0.000
LO	1	4.375	3.922	0.453 *	0.208	0.032
	2	3.660	3.192	0.468 *	0.217	0.033

Based on estimated marginal means: * The mean difference is significant at the 0.05 level; ^b^ Adjustment for multiple comparisons: Bonferroni.

**Table 4:** MANOVA test for German proficiency.

Dependent Variable	Satisfaction	German Proficiency Mean (1)	German Proficiency Mean (2)	Mean Difference (1–2)	S.E.	Sig. ^b^
LI	1	4.367	3.487	0.879 *	0.163	0.000
	2	3.984	2.897	1.087 *	0.156	0.000
CLI	1	4.429	3.406	1.023 *	0.171	0.000
	2	4.059	2.652	1.407 *	0.165	0.000
LO	1	4.375	3.660	0.715 *	0.217	0.001
	2	3.922	3.192	0.731 *	0.208	0.001

Based on estimated marginal means: * The mean difference is significant at the 0.05 level; ^b^ Adjustment for multiple comparisons: Bonferroni.

**Table 5:** Reliability analysis results.

Item	Corrected Item-Total Correlation	Cronbach’s Alpha If Item Deleted	Cronbach’s Alpha	Item	Corrected Item-Total Correlation	Cronbach’s Alpha If Item Deleted	Cronbach’s Alpha
PIN3	0.485	0.587	0.676	FL4	0.483	0.530	0.653
PIN4	0.496	0.572		FL5	0.462	0.559	
PIN7	0.485	0.587		FL7	0.446	0.581	
PN1	0.466	0.475	0.624	SA1	0.493	0.554	0.668
PN2	0.427	0.532		SA2	0.481	0.570	
PN3	0.405	0.563		SA3	0.464	0.592	
PIG1	0.450	0.531	0.636				
PIG2	0.447	0.537					
PIG3	0.439	0.547					

**Table 6:** Results of exploratory factor analysis.

Construct	KMO	Bartlett’s Sphere Test	Item	Commonality	Factor Loading	Eigenvalue	Total Variation Explained %
PIN	0.665	0.000	PIN3	0.603	0.776	1.821	60.710%
			PIN4	0.617	0.785		
			PIN7	0.602	0.776		
PN	0.641	0.000	PN1	0.616	0.785	1.712	57.061%
			PN2	0.564	0.751		
			PN3	0.531	0.729		
PIG	0.652	0.000	PIG1	0.586	0.765	1.737	57.893%
			PIG2	0.581	0.762		
			PIG3	0.570	0.755		
FL	0.655	0.000	FL4	0.616	0.785	1.773	59.086%
			FL5	0.589	0.768		
			FL7	0.567	0.753		
SA	0.661	0.000	SA1	0.618	0.786	1.803	60.088%
			SA2	0.603	0.777		
			SA3	0.581	0.762		

**Table 7:** Adaptation indices of the first-order CFA model.

Common Indices	χ^2^	df	χ^2^/df	RMSEA	GFI	AGFI	CFI	NFI	SRMR
Judgement criteria			<3	<0.08	>0.9	>0.9	>0.9	>0.9	<0.08
Value	81.686	80	1.021	0.006	0.979	0.969	0.999	0.962	0.024

**Table 8:** Convergence validity results.

Item	Factor Loading	t	S.E.	Sig.	CR	AVE
PIN3	0.63	14.076	0.034	0.001	0.675	0.410
PIN4	0.64	14.338	0.034	0.002		
PIN7	0.65	14.559	0.033	0.002		
PN1	0.65	14.591	0.035	0.002	0.628	0.362
PN2	0.60	13.189	0.036	0.001		
PN3	0.55	11.978	0.039	0.001		
PIG1	0.59	12.698	0.039	0.001	0.636	0.368
PIG2	0.62	13.440	0.039	0.002		
PIG3	0.61	13.238	0.037	0.001		
FL4	0.63	14.155	0.037	0.001	0.652	0.385
FL5	0.64	14.420	0.035	0.001		
FL7	0.59	13.257	0.036	0.001		
SA1	0.63	14.448	0.034	0.001	0.668	0.401
SA2	0.65	14.851	0.033	0.001		
SA3	0.62	14.235	0.035	0.002		

**Table 9:** Discriminant validity results.

	PIN	PN	PIG	FL	SA
PIN	0.640				
PN	0.549 **	0.602			
PIG	0.498 **	0.539 **	0.607		
FL	0.523 **	0.520 **	0.497 **	0.620	
SA	0.553 **	0.575 **	0.510 **	0.617 **	0.633

* *p* <, 0.05 ** *p* < 0.01 The diagonal number is the square root of the factor AVE.

**Table 10:** Adaptability of second-order CFA model.

Common Indices	χ^2^	df	χ^2^/df	RMSEA	GFI	AGFI	CFI	NFI	SRMR
Judgement criteria			<3	<0.08	>0.9	>0.9	>0.9	>0.9	<0.08
Value	18.177	24	0.757	0.000	0.992	0.986	1.000	0.982	0.018

**Table 11:** Adaptability of SEM.

Common Indices	χ^2^	df	χ^2^/df	RMSEA	GFI	AGFI	CFI	NFI	SRMR
Judgement criteria			<3	<0.08	>0.9	>0.9	>0.9	>0.9	<0.08
Value	83.610	84	0.995	0.000	0.979	0.970	1.000	0.961	0.025

**Table 12:** Direct and indirect effects.

Path	Direct Effect		Indirect Effect		Total Effect	
	β	B-C Sig.	β	B-C Sig.	β	B-S Sig.
INDE→FL	0.876	0.001	/	/	0.876	0.001
FL→SA	0.553	0.006	/	/	0.553	0.006
INDE→SA	0.436	0.095	0.484	0.005	0.920	0.001

**Table 13:** Moderating effect results.

Group	INDE→SA β		Nested Model Comparisons	
	Original Model	Specify PL β = Ilβ Model	CMIN	Sig.
PL	0.36	0.39	0.828	0.363
IL	0.83	0.41		

**Table 14:** Summary of survey results.

Studies	Content	Variables	Results
Prestudy	Select representative samples with high and low satisfaction.	SA	Select high satisfaction sample (BBAC), and low satisfaction sample (FY).
Study 1	Contrast AR picture books with traditional picture books in German instruction.	LI	Students who used AR picture books had higher interest in learning.
Study 2	Examine how AR picture books contribute to different levels of satisfaction regarding students’ learning.	LI, CLI, LO	AR picture books with higher satisfaction enhanced the learning of German.
	Examine how AR picture books help students with different levels of German proficiency.	LI, CLI, LO	AR picture books helped students with a higher level of German proficiency.
Study 3	Examine the variables that may affect students’ satisfaction with AR picture books in the structural equation model.	PIN, PN, PIG, INDE, FL, SA, PL	PIN, PN, and PIG constitute the second-order variable INDE; INDE directly affects FL; FL directly affects SA; INDE indirectly influences SA through FL mediation; PL does not play a significant moderating role between INDE and SA.

## Data Availability

Not applicable.
